# Effects of Respiratory Muscle Strength Training on Respiratory-Related Impairments of Parkinson's Disease

**DOI:** 10.3389/fnagi.2022.929923

**Published:** 2022-06-30

**Authors:** Jinyang Zhuang, Jie Jia

**Affiliations:** ^1^Department of Rehabilitation Medicine, Huashan Hospital, Fudan University, Shanghai, China; ^2^National Clinical Research Center for Aging and Medicine, Huashan Hospital, Fudan University, Shanghai, China; ^3^National Center for Neurological Disorders, Shanghai, China

**Keywords:** Parkinson's disease, respiratory muscle strength training, inspiratory muscle strength training, expiratory muscle strength training, rehabilitation

## Abstract

In addition to typical motor dysfunction, Parkinson's disease is also characterized by respiratory-related dysfunction. As a means of rehabilitation, respiratory muscle strength training (RMST) has been applied to restore Parkinson's disease (PD) functions. However, the current clinical value of RMST in the application for PD has not been widely established. This article aims to review the research progress of the application of RMST in PD rehabilitation to provide new sight into respiratory-related impairments management in people with PD.

## Introduction

Parkinson's disease (PD) is the second most common neurodegeneration disease, characterized by profound and selective loss of nigrostriatal dopaminergic neurons (Tysnes and Storstein, 2017; Abbas et al., 2018; Kip and Parr-Brownlie, 2022; Tansey et al., 2022). PD affects 6.1 million people worldwide (Collaborators GBDPsD, 2018; Armstrong and Okun, 2020), with more than 1% of 65-year-olds involved, and the prevalence will double by 2030 (Aarsland et al., 2021). The most typical characteristics of PD are motor impairments: bradykinesia, tremor, rigidity, and postural instability (Kalia and Lang, 2015; Pang, 2021). Besides, PD patients also suffer other impairments, such as dysphagia, with the prevalence varying between 11 and 87% (Takizawa et al., 2016; Schindler et al., 2021). Pneumonia, the leading cause of death in PD (Yoritaka et al., 2013; Won et al., 2021), is associated with dysphagia and affects respiratory function (Umemoto and Furuya, 2020). Reduced swallowing function, dyspnea, and decreased respiratory muscle strength even emerge in the early stage of PD, which often go undetected (Baille et al., 2018; Pflug et al., 2018).

A significant number of studies have revealed that respiratory muscle strength debility was related to poor respiratory function, dysphagia, coughing, and speech dysfunction for PD (Reyes et al., 2020a,b; Rodriguez et al., 2020; Valenza et al., 2020; Claus et al., 2021). Considering the respiratory muscle declines with increasing age and PD facilitates the trend (Sanches et al., 2014), taking measures to improve respiratory muscle functions is necessary. Respiratory muscle strength training (RMST) can improve respiratory muscle strength and may prevent aspiration pneumonia (Troche et al., 2010; Kuo et al., 2017; Claus et al., 2021). Emerging applications using RMST, such as speech rehabilitation, have also been proposed (Montero Ferro et al., 2019; Huang et al., 2020a, 2021; Reyes et al., 2020a,b; Cocks et al., 2022). *2014 European Physiotherapy Guideline for PD rehabilitation* suggested that RMST can improve respiratory-related disorders of PD (Keus et al., 2014). Systematic reviews about RMST have also indicated that RMST can be regarded as part of the PD rehabilitation program (Wang et al., 2019; Lopez-Liria et al., 2020; Rodriguez et al., 2020; van de Wetering-van Dongen et al., 2020). In the present review, we summarize the advanced research about RMST in the rehabilitation of people with PD, providing insight into respiratory-related impairments management in people with PD.

## Applications of Respiratory Muscle Strength Training

RMST aims to improve the force-generating capacity of respiratory muscle specifically. It is usually conducted using isocapnic hyperpnea trainers, incentive spirometers, and resistive and pressure threshold trainers (Sapienza, 2008). RMST contains inspiratory muscle strength training (IMST), expiratory muscle strength training (EMST), and a combination of IMST and EMST. RMST has been used widely in people with chronic respiratory diseases (Charususin et al., 2018; Xu et al., 2018; Figueiredo et al., 2020; Buran Cirak et al., 2022), and increased studies for RMST in neurodegenerative diseases are concerned (Huang et al., 2020b; Valenza et al., 2020; Dorça et al., 2021). RMST is easy to perform and cost-effective for older adults with PD, which can be used in clinical or at home to improve respiratory-related dysfunctions.

### Respiratory Muscle Strength

Respiratory impairments of the central nervous system in neurodegenerative disorders, including PD, are manifested by a restrictive ventilation pattern and decreased inspiratory and expiratory pressure, directly associated with the decline in respiratory muscle strength (Reyes et al., 2013; Docu Axelerad et al., 2021; Guilherme et al., 2021). The drop in respiratory muscle is apparent even in the early stage of PD (Santos et al., 2019). Besides, a sedentary lifestyle and reduced physical activity in PD may also contribute to respiratory muscle weakness (Aktar et al., 2020). The application of RMST in people with PD is targeted firstly at improving respiratory muscle strength.

In 2005, Inzelberg et al. recruited 20 PD patients (Hoehn-Yahr scale, stage II ~ III). They used IMST with beginning breathing at a load equal to 15% of their maximal inspiratory mouth pressure for 1 week, increasing gradually as an experimental intervention. It was revealed that IMST could improve inspiratory muscle strength and dyspnea in PD patients (Inzelberg et al., 2005). Saleem et al. performed EMST in 20-week home treatment for early idiopathic PD patients. Compared to before treatment, the study found that the expiratory muscle strength improved significantly after 20-week EMST (Saleem et al., 2005). Silverman and other scholars showed that respiratory muscle weakness was common in moderate to severe idiopathic PD patients and reported that using RMST could enhance maximum expiratory pressure (MEP) (Silverman et al., 2006). Kuo et al. divided 13 outpatients with PD (Hoehn Yahr scale, stage I ~ III) into 3 days/week of EMST, 5 days/week of EMST, and control groups (Kuo et al., 2017). In both experiment groups, 75% MEP was used as training load, and in the control group, 0% MEP was used as training load. The results revealed that either 5 days/week or 3 days/week of EMST increased expiratory muscle strength. Huang et al. found that RMST increases maximal inspiratory pressure (MIP) and MEP, indicating that RMST could improve respiratory muscle strength for PD (Huang et al., 2020a). Recently, Oguz et al. designed a combination treatment that combined RMST and walking training. The RMST using the threshold loading trainer contained IMST and EMST. Researchers conducted a randomized controlled study to compare the combination intervention with a single RMST. The results revealed that a combination intervention could further facilitate the MEP than a single RMST (Oguz et al., 2022).

As described above, RMST can improve the respiratory muscle strength of PD. However, the conclusion may be cautious because the MEP and MIP values have a learning effect on evaluating respiratory muscle strength (Smeltzer and Lavietes, 1999; Reyes et al., 2020a,b). In addition, the RMST protocol for respiratory muscle strength varies in different studies, so we can't make specific training doses.

### Cardiorespiratory Function

Cardiorespiratory function decline is also a frequent non-motor impairment of PD and is associated with limited mobility. Compared to cardiovascular autonomic nervous function, the application of RMST in pulmonary rehabilitation is more frequent. This may be because respiratory muscle is directly related to pulmonary function. A clinical trial found that IMST did not improve spirometry parameters, including forced vital capacity (FVC) and forced expiratory volume in 1 s (FEV1) (Inzelberg et al., 2005). Reyes et al. pointed out that slow vital capacity (SVC) and FVC did not improve in expiratory and inspiratory groups compared with control groups (Reyes et al., 2018). Sapienza et al. also found EMST had no additional effect on maintaining the spirometry parameters, including forced expiratory volume in 1 s/forced vital capacity (FEV1/FVC), FVC, FEV1, and peak expiratory flow (Sapienza et al., 2011).

The autonomic nervous system plays a vital role in the lung ventilation regulation, gas exchange, and smooth muscle function of the airway (Herer et al., 2001). Recently, some researchers have concentrated on the simultaneous effects of RMST on lung and cardiovascular autonomic nervous function. Alyne Montero Ferro designed the study protocol to investigate the impact of IMST on the cardiac autonomic nerve and pulmonary function in 26 PD patients (modified Hoehn Yahr scale, Stage I ~ III) (Montero Ferro et al., 2019). Huang et al. recruited 75 individuals with PD. They assigned them to the RMST and control groups (Huang et al., 2020a). All patients received 30-min training twice a day, at least 5 days a week for 12 weeks. The results showed that RMST could improve MIP, MEP, and heart rate response to deep breathing (HRDB) parameters, one of the indicators of cardiovascular autonomic nerve function.

As far as we know, RMST has no noticeable effect on spirometry parameters (FEV1/FVC, FVC, FEV1, SVC) in PD (Inzelberg et al., 2005; Sapienza et al., 2011; Reyes et al., 2018; Huang et al., 2020a). Interestingly, the effects of RMST on cardiovascular autonomic nerve function in people with PD have attracted attention and made progress. Further studies in RMST should be conducted to determine its efficacy and mechanism in the cardiorespiratory function of PD.

### Swallowing Function

Swallowing deficit exists in earlier stages of PD and impacts 80% of PD patients during the process of their disease (Kalf et al., 2012; Pflug et al., 2018). During the swallowing process and the cricopharyngeal muscle opening, the suprahyoid muscle makes the hyoid bone anterior-superior movement to facilitate airway protection (Pearson et al., 2011). The weakness of the suprahyoid muscle causes the inadequate anterosuperior movement of the hyoid bone, dysphagia, and aspiration pneumonia (Pearson et al., 2012). The latter is one of the major causes of death in PD patients in the advanced stages (Fernandez and Lapane, 2002; Hegland et al., 2014).

EMST has been shown to increase and prolong activation of the suprahyoid muscle group (Wheeler-Hegland et al., 2008) and may affect bradykinesia of swallowing, a hallmark of PD-related dysphagia (Suttrup and Warnecke, 2016; Dziewas et al., 2019). Pitts et al. recruited 10 PD patients aged 60-82 (Hoehn Yahr scale, stages II-III) for 4 weeks of EMST, 5 days a week, 5 groups a day, five times a group. The researchers set the resistance as 75% MEP and evaluated the degree of penetration/aspiration (P/A). The study found that the P/A of patients decreased significantly after treatment (Pitts et al., 2009). Troche et al. randomly assigned 60 PD patients (Hoehn Yahr scale, phase II-IV) to the EMST group and sham EMST group (Troche et al., 2010). The training load in the EMST group was set to 75% MEP every week for 4 weeks, 5 days a week, 20 min a day. The study showed that EMST improved swallowing safety and glossopharyngeal activity during swallowing. Claus and other scholars found that the pharyngeal residue of PD patients was significantly reduced after EMST (the training does: as Troche et al.) (Claus et al., 2021). Although researchers discovered no change in the swallowing-related cerebral cortex by the whole brain magnetogram (Claus et al., 2021), it is necessary to further explore the central and peripheral mechanisms of EMST on swallowing function. Byeon proposed the simultaneous combination of the postural techniques and EMST (Byeon, 2016) and found that compared with single EMST, the simultaneous training improved the dysphagia of PD more obviously.

Following the above studies, EMST positively affects swallowing deficits of people with PD. A consensus on treating dysphagia of PD has also indicated that EMST could benefit swallowing function and airway protection (Schindler et al., 2021). The intensity of the initial training of EMST is typical to set at 75% MEP and increases gradually. Other similar EMST regimes contain 5 days a week for 4 weeks in the above studies (Pitts et al., 2009; Troche et al., 2010; Claus et al., 2021). In addition, EMST intervention does not cover all swallowing processes and all stages of Parkinson's disease (PD), especially late-stage PD, limiting its clinical applicability.

### Cough

Regular coughing is a protective response that helps expel foreign bodies and mucus from the airway in the chest. The contraction involves the coordinated respiratory and intrinsic laryngeal muscles facilitating cough, so the decline of cough ability is also related to respiratory muscle weakness (Widdicombe et al., 2011). For example, the decreased expiratory muscle strength may weaken the ability to generate expiratory pressure and lead to less effective coughing (Ruoppolo et al., 2013).

Many studies have investigated RMST on the cough capacity of PD. Pitts et al. found that cough airflow velocity was improved during voluntary cough after using EMST (Pitts et al., 2009). Reyes et al. randomly assigned 31 PD patients to the IMST, EMST, and control groups (Reyes et al., 2018). The training regime was six times a week for 2 months, with five rounds of training each time and five repetitions each round. The study found that EMST can improve the voluntary cough peak flow rate compared with the other two groups, and EMST had more advantages in improving cough function than IMST. Sapienza et al. showed that after 4 weeks of EMST, the duration of the compression period and expiratory rise time of PD patients were significantly shortened, and the amount of cough increased considerably (Sapienza et al., 2011). Reyes et al. combined EMST with air stacking (AS) and compared the effect on cough function among EMST with AS, EMST, and control groups (Reyes et al., 2020a). The study found that combination treatment was more effective than EMST in improving reflex and voluntary peak cough. The combination treatment had a more significant effect on voluntary cough than reflex cough.

The above studies show that EMST can increase cough airflow and improve voluntary peak cough flow. EMST with AS may be a more robust treatment for cough recovery. Even though EMST has been shown to enhance cough ability in patients with PD, the conclusion should be cautious because of the small sample size and relatively poor study quality (Reyes et al., 2018, 2020a). A further large sample and high-quality studies should be conducted to explore the effects of RMST on the cough capacity of PD.

### Speech

In people with PD, impaired lung function may be concerned with the pathophysiology of axial symptoms such as dysarthria (Hammer, 2013). People with PD frequently experience respiratory deficits, such as weak and rigid respiratory muscles, chest wall stiffness, and bradykinesia of abdominal muscles. Due to these abnormalities, vocal intensity is affected by reduced pulmonary capacity and airflow required to vibrate the vocal folds (Irzaldy et al., 2016; Hassan et al., 2018). By increasing exhalation and inhalation volume, enhanced respiratory muscle strength may result in more significant positive intrathoracic pressure and a greater flow of air to stimulate voice production (McCool, 2006; McCool and Rosen, 2006). Based on the relationship between respiratory muscles and speech, RMST has been used as a voice treatment (Ray et al., 2018; Belsky et al., 2021; Desjardins et al., 2022; Perin et al., 2022).

RMST has been used for speech rehabilitation in individuals with PD. Darling-white et al. recruited 12 PD patients for EMST using 75% MEP and weekly adjustments over 4 weeks. The study found that people used more typical lung volume for speech after EMST, indicating that EMST improved speech breathing in PD patients (Darling-White and Huber, 2017). Reyes et al. used home-based IMST and EMST as experimental interventions and measured the volume of voice produced by PD patients (Reyes et al., 2020b). The study revealed that IMST increased the maximum volume grew, and EMST improved the peak subglottic and sound pressure levels.

RMST may be a valuable treatment for the speech rehabilitation of PD. Despite this, it is worth noting that RMST studies in PD patients with speech disorders are still relatively small compared to other functions, and more investment in this field is needed.

## Detraining Effect

An effect of detraining is the partial or complete loss of training-induced adapations, due to an insufficient stimulus (Mujika and Padilla, 2000a,b). Like other skeletal muscles, respiratory muscles may experience a decline in strength during a detraining period (Romer and McConnell, 2003; Baker et al., 2005). Romer and McConnell showed that six healthy subjects experienced detraining effect after receiving a 9-week IMST. During 9 weeks after the intervention, inspiratory muscle function declined more and plateaued between 9 and 18 weeks, remaining above pre-IMST values (Romer and McConnell, 2003). Baker et al. compared the detraining effect of 4-week EMST to 8-week EMST in healthy participants, indicating that the MEP was reduced by 7 and 10%, respectively, at the 8th week of the detraining period (Baker et al., 2005). Moreover, MEP did not significantly differ between groups during detraining. In chronic neuromuscular diseases, the respiratory muscles determine the subjects' vital capacity, and their deterioration can result in inadequate ventilation, ineffective coughing, and dysphagia (Laveneziana et al., 2019; Galea, 2021; Brennan et al., 2022).

The detraining effect of RMST in people with PD has drawn attention. In a case report, Saleem et al. showed that MEP improved by 158% compared with baseline at the end of 20 weeks of EMST. It was found that MEP decreased by 16% in 4 weeks after the cessation of treatment, but improvement is still evident compared to before treatment (Saleem et al., 2005). Troche et al. observed the clinical efficacy of 10 PD patients in 3 months following a 4-week EMST (Troche et al., 2014). They found that MEP increased by 19% after 4 weeks of EMST, and after the 3-month post detraining, MEP decreased by 2% but was still higher than the baseline value of 17%. There was no change in the improvement of swallowing safety from the end of treatment to post-detraining. Claus et al. found that individuals with PD can maintain the swallowing function improvement for at least 8 weeks after receiving a 4-week EMST (Claus et al., 2021). In addition, Huang et al. pointed out that after stopping the 3-month RMST, the improvement of respiratory muscle strength in PD patients lasted for at least 6 months, while the impact on cardiovascular autonomic function was <6 months (Huang et al., 2021).

As described above, RMST has a lasting impact on respiratory muscle strength, swallowing function, and cardiovascular autonomic function for PD. Considering that the effect of RMST decreases after intensive training, it is essential to design detraining prevention which refers to limiting and counteracting detraining effects through setting training strategies (Troche et al., 2014; Girardi et al., 2020; Huang et al., 2021). The detraining prevention of RMST in people with PD has not been studied yet, which will become a newly explored area.

## Conclusion

RMST has been used to treat decreased respiratory muscles, dysphagia, ineffective coughing, speech disorders, and cardiorespiratory dysfunction in people with PD. A lasting impact on respiratory muscle strength, swallowing function, and cardiovascular autonomic function can be observed after stopping RMST. RMST-based combination therapy is expected to be a meaningful treatment initiative for PD patients. Indeed, RMST still has some limitations in the application for PD rehabilitation. Firstly, the training regimes (frequency, intensity, type, and time) of RMST vary in most studies. As described above, a more consistent and positive protocol can be found in the application of EMST for swallowing deficits, but not for other functions. Secondly, fewer RMST studies focused on patients with advanced or late-stage PD who had more apparent non-motor impairments such as dysphagia. Thirdly, the mechanism of RMST is still not elucidated. Because of the above limitations, we anticipate a more significant number of high-quality, multicenter randomized controlled trials will be conducted to promote the use of RMST.

## Author Contributions

JZ and JJ contributed to the conception and design of the study. JZ wrote the first draft. Both authors contributed to the article and approved the submitted version.

## Funding

This work was supported by the National Key R&D Program of China (Grant No. 2018YFC2002300 and 2018YFC2002301), National Natural Science Foundation for Innovative Research Group Project of China (Grant No.82021002), and National Natural Science Foundation of China Major Research Program Integration Project (Grant No.91948302).

## Conflict of Interest

The authors declare that the research was conducted in the absence of any commercial or financial relationships that could be construed as a potential conflict of interest.

## Publisher's Note

All claims expressed in this article are solely those of the authors and do not necessarily represent those of their affiliated organizations, or those of the publisher, the editors and the reviewers. Any product that may be evaluated in this article, or claim that may be made by its manufacturer, is not guaranteed or endorsed by the publisher.
